# Composites cement/BaSO_4_/Fe_3_O_4_/CuO for improving X-ray absorption characteristics and structural properties

**DOI:** 10.1038/s41598-022-23908-0

**Published:** 2022-11-10

**Authors:** Muh. Syahrial Gharissah, Ardiansyah Ardiansyah, Sitti Rahmah Pauziah, Nurul Awaliyah Muhammad, Roni Rahmat, Heryanto Heryanto, Dahlang Tahir

**Affiliations:** Department of Physics, Hasanuddin University, Makassar, 90245 Indonesia

**Keywords:** Medical research, Materials science, Applied physics

## Abstract

Composite cement/BaSO_4_/Fe_3_O_4_/CuO with a thickness of 0.6 cm for various amounts of CuO: 2 wt%, 4 wt%, 6 wt%, and 8 wt% were successfully synthesized for the X-ray radiation shield. The bonding characteristics of composite and structural properties were determined using Fourier transform infrared spectra for the wavelength range of 4000–400 cm^−1^ and X-ray diffraction with the range of 2θ from 25° to 50°, respectively. The shielding ability was measured using a mobile X-ray with an energy of 55, 66, and 77 keV for determining the mass and linear attenuation coefficient, electronic and atomic cross-section. These shield characteristics best agreement with theoretical calculation from the XCOM database for energy < 77 keV with half value layer (HVL) < 0.3 cm. The best shielding in this study indicated by the lowest HVL and MFP is composite for CuO 8 wt%. The HVL and MFP shows better values compared to the previous reported using composite rubber-based, indicated high potentials composite in this study for design new and efficient radiology rooms as an alternative concrete, especially for X-ray radiation, in the future.

## Introduction

Radiodiagnostics is a branch of radiology in medicine that plays an active role in medical examinations and provides information to the doctor about a patient’s condition to decide on the subsequent treatment. Radiology is the primary diagnostic tool responsible for radiation exposure from sources that can ionize the matter^1^. In the radiology room, many factors affect the effectiveness of the shielding material used to protect people working in the radiation facilities, such as radiation energy, type of radiation, the thickness of the material, the denser material, and the effective material against harmful X-rays^2^. So, the patient and the workers in the radiology room need protection from radiation exposure^3^.

One of the severe problems that can affect human health is radiation leakage, where X-ray radiation emits energy from metallic sources^4,5^. The most common dangerous forms of radiation are X-rays, gamma rays, and neutrons because they have a high penetrating ability and enough energy to ionize the matter^6–8^. These form of radiation has significant effects detrimental to humans for a period that will suffer from genetic and blood cell damage^6,9^. Gamma rays penetrate far more significantly than alpha and beta rays which cause genetic mutations and damage the structure of cellular living organisms^10,11^.

In living cells, possible effects of ionizing radiation such as gamma rays and X-rays require strong radiation protection. High-density materials such as lead and concrete for protection from radiation must be high-density materials, which can act as barriers and reduce the effect of radiation^7^. Still, most scientists now think of new materials with environmentally friendly concepts. Therefore, new materials with high protection are required for radiation workers and patients from exposure to minimize the effects of radiation and avoid radiation risks^12^.

A shield of radiation is a material that absorbs radiation to protect people from the harmful effects of high-energy photons widely used in industry, research, and medical applications^13–15^. The radiation shields materials reported such as concrete, alloys, glass, tiles, and clay bricks^15–19^. The shielding materials must have high density, good radiation attenuation, and low toxicity in manufacturing^15,16^.

Traditional dose reduction methods by Pb shields are widely used to reduce external radiation exposure doses of X-rays or γ-rays^20,21^. Lead has a very good radiation shield and very popular as a low cost and high efficiency for X-rays or γ-rays radiation shielding. The performance lead supported by the high atomic number and mass density which are required for an effective photon shield. However, the intoxication of Pb in the human body and to the generation of secondary waste during the disposal of Pb has encourage researcher to find alternative materials for shield^22,23^. There are two possible ways of reducing Pb toxicity level: by mix with other materials and use Pb-free shielding in the form of nanocomposite materials. For reducing Pb toxicity some reseacher was reported composite using natural polymer such as cement-based with additional Pb shows HVL of 0.004 cm^−1^ for 80 keV^24^ and filler fly ash and sand with additional glass shows HVL of 0.11 cm^−1^ for 140 keV^25^. Materials for Pb-free shielding was reported from various researcher with the composition ratio of various polymers and filler with high-density metals or metal oxide^23,26^. These studies demonstrated that the performance of Pb-free shielding with linear attenuation coefficients improved because of high atomic number and high density which suitable for medical applications specially shiled radiation^27–29^.

Performance of various materials for Pb-free shielding were compared with that of Pb shielding. For Pb-free shielding shows excellent radiation shielding rate and deduce the bremsstrahlung radiation^30^. The composite polymer-based for shield by using natural rubber filled with Bi_2_O_3_ and more complex filler using acetates/ carboxylates/ BiF3/ ThF_4_/ metal/ fluorides shows HVL 0.2 cm^−1^ for energy 50 keV and HVL 0.21 cm^−1^ for energy 45 keV, respectively^31,32^. The composite mixed Pb with some natural polymer shows very good for high energy (> 100 keV) and some composite Pb-free shows very good for low energy (< 50 keV) but most of them have poor shielding effects against middle-energy range for gamma rays and diagnostic X-rays^33,34^.

The below reported radiation shield uses BaSO_4_ and Fe_3_O_4_ for aprons, anti-radiation concrete, and bioactive glass. The BaSO_4_ show quickly processed and similar protective ability to lead due to a high photon attenuation coefficient (linear and mass attenuation coefficient), effective atomic number, and good electron density. Based on these specifications, BaSO_4_ would be an excellent choice to protect against radiation^35–37^. As for Fe_3_O_4_, it is good to increase the attenuation of electromagnetic waves, both electrical and magnetic properties^38^.

Meanwhile, CuO has a high absorption efficiency, is environmentally friendly, and is low cost, with some interesting electronic properties: small bandgap and p-type conductivity^39^. Ref.^40^ reported that, the designing and developing lightweight shields using CuO with performance efficiency superior, more than 99.99% in attenuating electromagnetic wave. They used composite polymer/CuO with single layer and hybrid system with high protection as a shield. In our previous publication showed that CuO high porosity, low coercivity and shows very good as an absorber electromagnetic wave^41^. These attributes included: ease production and environmentally friendly (since they can be recycled). The chemical composition can be modified when added with other materials to become composite through simple methods such mechanical alloying and other easy chemical and physical processes. These reasearch were reported that, the potential of natural or synthetic polymer as a matrix and BaSO_4_, Fe_3_O_4_, and CuO, metal, and fluorides as a filler for shield. Now is the research era where the scientist consent to the evironment effect means that should be using environmentally friendly materials for various applications. In our previous research was reported that the structural properties from analysis XRD and FTIR spectra for high amount of BaSO_4_/Fe_3_O_4_ in composite cement/BaSO_4_/Fe_3_O_4_ is unstable^27^. We showed that high stability structural properties for low concentration (5% BaSO_4_/Fe_3_O_4_) in composite indicated by the distance between the wavenumber of transversal and longitudinal optical phonon vibration mode is high even after irradiation. Therefore, for composite in this study, we used natural polymer (cement) and keep constant low concentration BaSO_4_/Fe_3_O_4_ with various concentration of CuO as a filler to form composite Cement/BaSO_4_/Fe_3_O_4_/CuO for eco-friendly materials. This issue has not been studied yet. The search references of composite Cement/BaSO_4_/Fe_3_O_4_/CuO shows no reported for various amounts of CuO for shielding application and compared the absorption characteristics with theoretical calculation from the XCOM database. Hence, in this study, we use CuO to increase X-ray absorption efficiency combined with BaSO_4_ and Fe_2_O_3_ in composite Cement/BaSO_4_/Fe_3_O_4_/CuO for a medium energy radiation shield to provide alternative concrete for the efficiency and development of a new radiology room. The concrete was made by mixing Portland cement with barium sulfate (BaSO4), iron (II, III) oxide (Fe_3_O_4_), and copper (II) oxide (CuO). The shielding performance was tested using a mobile X-ray to determine the mass and linear attenuation coefficient. We continue determining absorption characteristics; atomic and electronic cross-section, half-value layer, and mean free path. Fourier transforms infrared (FTIR) spectra analyzed the bonding and functional group and structural properties from X-ray diffraction (XRD). We compared some of these absorption characteristics with theoretical calculations from the national institute of standards and technology (NIST in the XCOM database). Software such as XCOM usually used for theoretical calculation of physical phenomena when the incoming radiation travel inside the material radiation shielding and have interaction with atom and electron^42,43^.

## Materials and methods

### Materials

Portland cement (PC) was supplied from the local company (Semen Tonasa, Indonesia). Barium Sulfate (BaSO_4_) with purity 99% and particle size < 100 nm, Iron (II, III) Oxide (Fe_3_O_4_) with purity 99.5% and average particle size 20 nm, and Copper (II) oxide powder (CuO) with particle size is < 10 μm and assay 98% were supplied from Merck.

### Sample preparation

The PC powder, BaSO_4_, CuO, and Fe_3_O_4_ are mixed together to form composites concrete. There are four types of samples and the compositions details for every samples as shown in Table 1. The name of samples based on the amount of CuO in composites for example PC/BaSO_4_/Fe_3_O4/CuO (PBFC (CuO 2 wt%)) for 2 wt% CuO.

**Table 1:** Chemical weight compositions of concrete cement/BaSO_4_/Fe_3_O_4_/CuO material for various amount of CuO as an X-ray shielding.

Chemical	Composition (wt%)			
	PBFC (CuO_2_ wt%)	PBFC (CuO_4_ wt%)	PBFC (CuO_6_ wt%)	PBFC (CuO_8_ wt%)
PC	88	86	84	82
BaSO_4_	5	5	5	5
Fe_3_O_4_	5	5	5	5
CuO	2	4	6	8

The synthesizing methods of composites are divided by three step: first, mixing PBFC and CuO using a magnetic stirrer at a constant speed of 500 rpm for 30 min, then dropwise slowly 40 ml distilled water while continue stirring to form paste. The paste was poured into the mold with the size of (10 × 10 × 6) cm, then allowed for 24 h at room temperatures. Finally, the dried samples are keep at room temperature for 28 days in a closed container for further use.

### Characterization

The X-ray irradiation using mobile X-rays (Multimobile 2.5) that has an energy of (1–120 keV) but in this study only used the energy of 55–77 keV as usually used for diagnostic purposes in hospital by detector X-ray multimeter (Ray Safe). The irradiation process is carried out in the Health Facility Safety Center (BPFK) Makassar, Indonesia. The FTIR spectrometer type (Shimadzu Corp), measurements were set up at the range of the wavelength 4000–400 cm^−1^ to determine the bonding characteristics. The X-ray diffraction spectroscopy (XRD) type (Shimadzu 7000), measurements were set up at 2θ from 25° to 50° to determine the structural properties of composites.

## Result and discassion

### X-ray diffraction (XRD)

**Figure 1:**
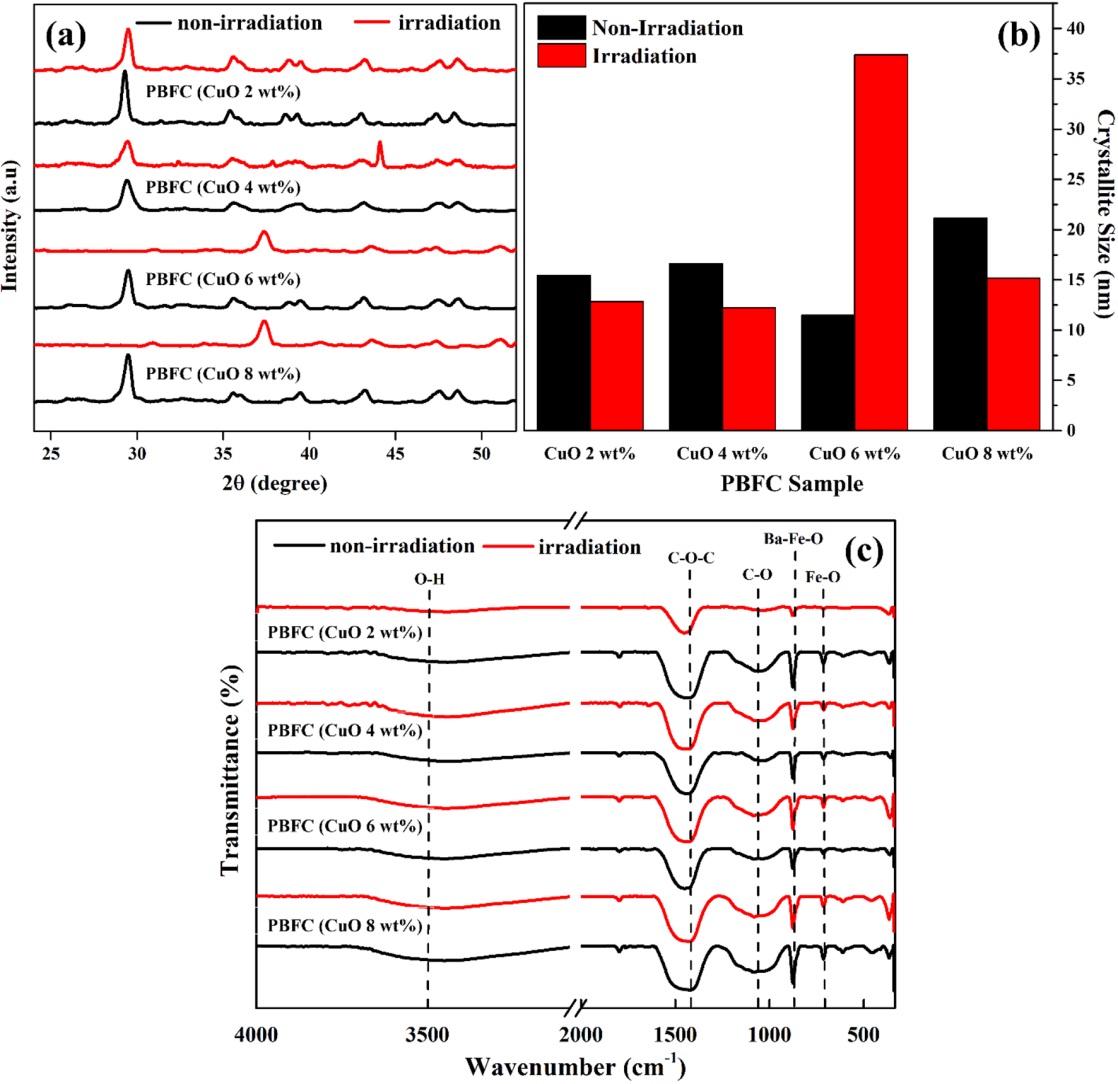
(**a**) X-ray diffraction (XRD) spectra, (**b**) Crystallite Size determined from XRD spectra, and (**c**) Fourier transform infra-red (FTIR) spectra of composites PBFC for various amount of CuO in composite for irradiation and non irradiation.

Figure 1a shows XRD spectra of PBFC for various amount of CuO and for irradiation treatment and non irradiation. For PBFC with 2 wt% and 4 wt% of CuO, the diffraction peak 2θ around 26°–30° after irradiation disappears. For diffraction peak 2θ at 35.54° and 39.41° shows converges after irradiation, produce single diffraction peak 2θ at 37.42°. These phenomena at low composition of CuO (≤ 4 wt%) indicated that the atoms are unstable when exposed to radiation, some of the atom break the bond with binder (cement) and moves or come out from the atomic structure of composite^44^.

The intensity of the diffraction peak 2θ at 29.43° for PBFC (CuO 6 and 8 wt%) shows decreased and the FWHM was increased. The intensity decrease with increasing the FWHM will affected to the crystallite size as can be seen in Fig. 1b. The crystallite size for non irradiation higher than that of irradiation composite PBFC for CuO 2, 4, and 8 wt% due to the stress behavior of crystal structure during the irradiation process^45^. For CuO 6 wt% shows higher crystallite size for irradiation may due to aggolmeration of CuO in composite. The average crystallite size was calculated by Debye–Scherrer equation^46^:

1 $$ D = \frac{{K\uplambda }}{\beta \cos \theta } $$

where (*D*) is a crystallite size, $\left( K \right)$ is a shape factor which is usually 0.9 for spherical particles, $\left(\uplambda \right)$ is the wavelength radiation of X-Ray (Cu source), $\beta$ is full width at half the maximum (FWHM).

### Fourier transform infra-red

The FTIR spectra of four samples in this study (Fig. 1c) shows full absorption spectra in the wavenumber between 400 and 4000 cm^−1^. The wavenumber at 707 cm^−1^ shows the typical absorption of Fe–O bonds^46^. The characteristic bands at 877 cm^−1^ was identified come from the Ba–Fe–O bond and at 1055 cm^−1^ is ascribed to the C–O bond^47^. The very small peak at the wavenumber of 1740 cm^−1^ was identified stretching of the C=O group and the broad and pronounced peak at the wavenumber of 1437 cm^−1^ indicated C–O–C stretching CH_3_ bending vibrations^1,48^. The absorption bands in the wavenumber at 3500 cm^−1^ are attributed to the O–H stretching vibration of adsorbed water molecules^44^.

### Absorption performance

The radiation properties were determined from the data of X-ray mobile of composites PBFC for various amount of CuO in composite with the thickness of 0.6 cm for an energy is 55 keV, 66 keV, and 77 keV. The equation of Beer-Lambert’s law used for calculation the linear attenuation coefficient (LAC) (μ) and mass attenuation coefficient (MAC) (μ_*m*_) as follows^49^:

2 $$ I = I_{o} e^{ - \mu x} $$

3 $$ \mu_{m} = \frac{1}{\rho x}\ln \left( {\frac{{I_{o} }}{I}} \right) $$

where $$I_{o} $$ and $$I$$ are the initial intensity and intensity after irradiation, $$x$$ is the thickness of the composites PBFC and $$\rho$$ is the density of the composite PBFC.

#### Linear attenuation coefficient (LAC) and mass attenuation coefficient (MAC)

**Figure 2:**
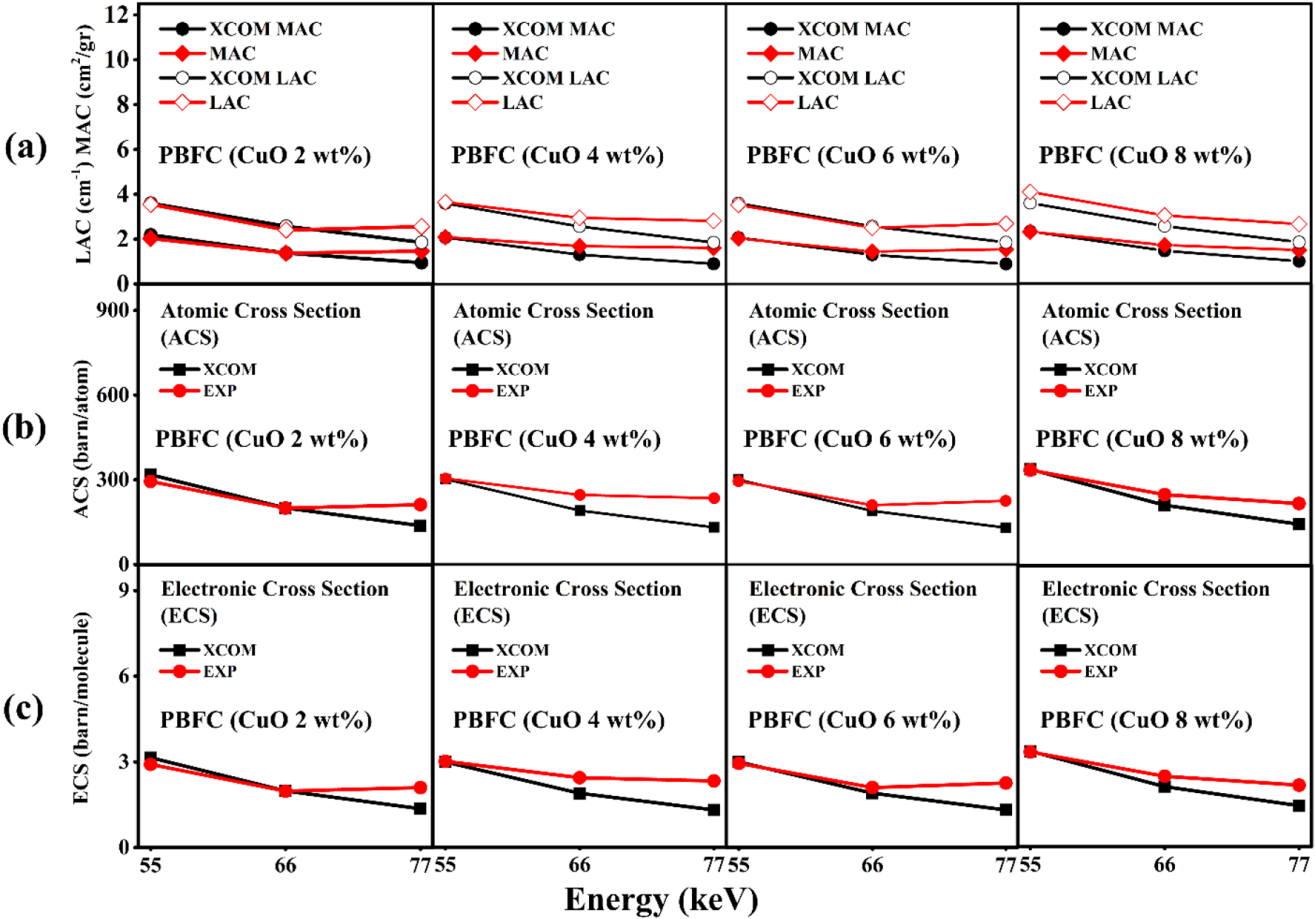
The linear (LAC) and mass attenuation coefficient (MAC) (**a**), atomic cross section (ACS) (**b**), and electronic cross section (ECS) (**c**) of shielding PBFC for various amount of CuO in composite for energy 55, 66, and 77 keV. We have included the theoretical calculation (XCOM) database for comparison.

Figure 2A shows LAC and MAC of PBFC, for energy 77 keV the experiment data is higher compared with that of theoretical calculation (XCOM), similar trend for all PBFC in this study. For photon beams when they are entering the materials will loses energies in many ways, may vibration of atom result electron hoping, electron migration, scattering of atoms, and some of these energies converted to the thermal energy^27,50–52^. These phenomena depend on the structure of materials, for the theoretical calculation (XCOM) database use the uniform crystal structure in their calculation, which may be different in the experimental conditions^53^.

#### Atomic cross section and electronic cross section

**Figure 3:**
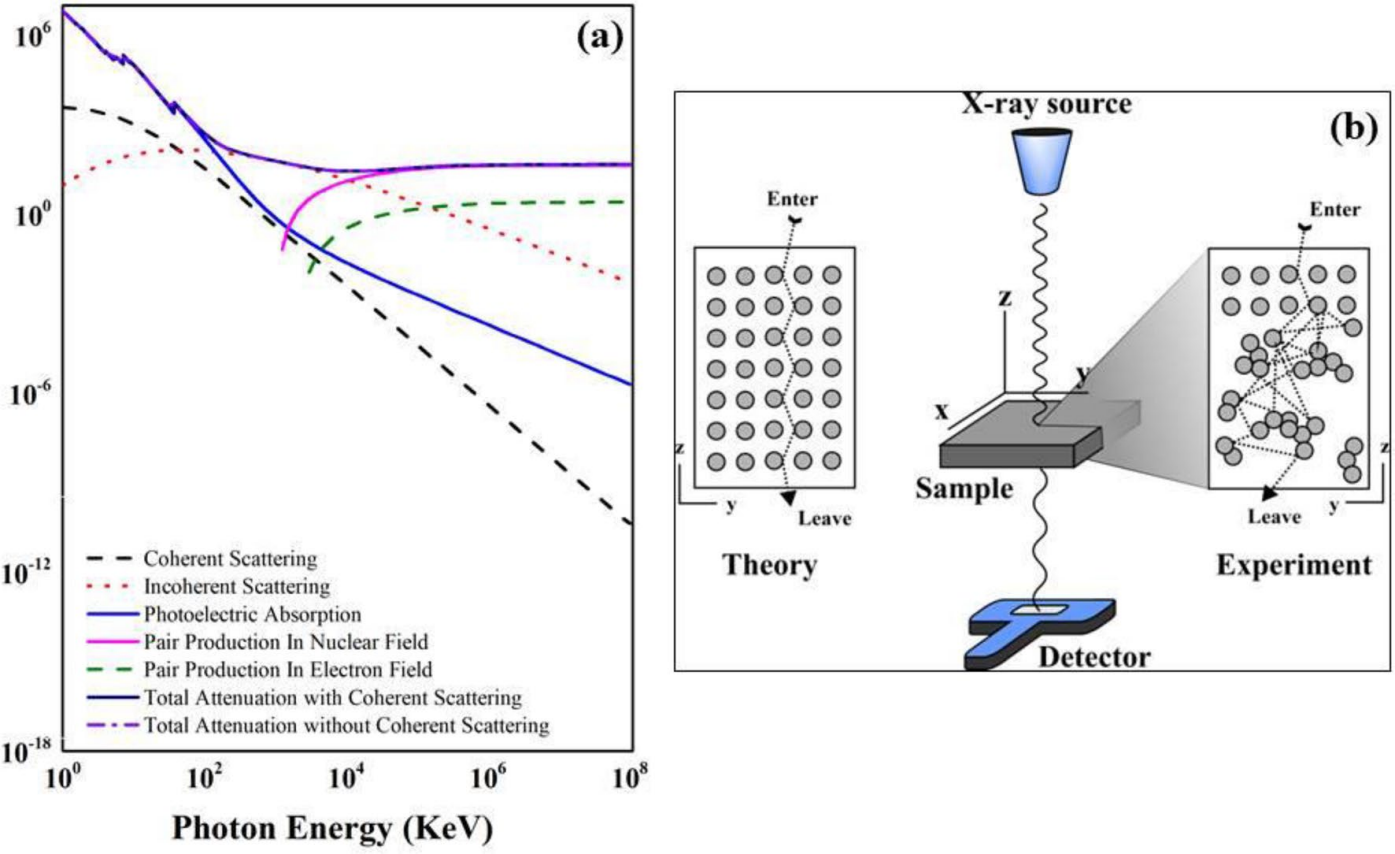
(**a**) XCOM full spectra of PBFC and (**b**) schematic illustration of PBFC composites when the photon incoming to the composite for theoretical based (theory) with uniform structure and for experimental condition with non-uniform structure.

Figure 2B, c are for ACS and ECS were calculated using the mass attenuation coefficient obtained from experimental values and theoretical calculation (XCOM) database^43^. ACS and ECS shows very good agreement with theoretical calculation at low energy and decrease with increasing the irradiation energy for theoretical calculation but for experimental increase with increasing the irradiation energy, similar trend for all composite^54^. The schematic illustration when the X-ray entering the PBFC composite for theoretically (XCOM) condition compared with experimental results in this study (Fig. 3b). For higher energy (≥ 77 keV) shows all absorption characteristics from the experimental data in this study is higher compared with that of theoretical calculation (XCOM) which may be due to the agglomeration below the surface atoms of the samples as illustrated in Fig. 3b. The photon travels inside composite deeper, may hit the agglomeration of various atoms, another side of agglomeration is pore which use as a trap for the X-ray consequently increase the absorption ability^55^. For low energy (< 77 keV) the value between experiment and theoretical by XCOM shows best agreement due to the photon hit only the atoms at the surface which having uniform arrangement. The value of the MAC can be used to determine of ACS and ECS using the following equation:

4 $$ \left( {\upsigma _{{{\text{t}},{\text{a}}}} } \right) = \frac{1}{{{\text{N}}_{{\text{A}}} }}\sum\limits_{{\text{i}}} {{\text{f}}_{{\text{i}}} {\text{A}}_{{\text{i}}} \left( {\upmu _{{\text{m}}} } \right)_{{\text{i}}} } $$

5 $$ \left( {\upsigma _{{{\text{t}},{\text{el}}}} } \right) = \frac{1}{{{\text{N}}_{{\text{A}}} }}\sum\limits_{{\text{i}}} {\frac{{{\text{f}}_{{\text{i}}} {\text{A}}_{{\text{i}}} }}{{{\text{Z}}_{{\text{i}}} }}\left( {\upmu _{{\text{m}}} } \right)_{{\text{i}}} } $$

where ${\text{f}}_{{\text{i}}} = {\text{n}}_{{\text{i}}} /\sum\nolimits_{{\text{i}}} {{\text{n}}_{{\text{j}}} }$ are the fractional abundance, A_i_ is the atomic mass of the composite, and Z_i_ is the atomic number of the composite.

### Half value layer (HVL) and mean free path (MFP)

Table 2 shows HVL and MFP which is important quantities that describe the effectiveness of radiation shielding^56^. HVL represents the thickness of the sample which reduces the initial intensity of the photons by half, and MFP is the distance between two collisions when the photon traveled before scattering or absorption occurs^57,58^. The lower HVL and MFP value indicated good in absorbing radiation of the shield, which determined by^59,60^:

6 $$ HVL = \frac{\ln \left( 2 \right)}{\mu } = \frac{0.63}{\mu } $$

7 $$ MFP = \frac{1}{\mu }. $$

**Table 2:** Half value layer (HVL) and mean free path (MFP) of PBFC for various amount of CuO in composites.

Energy (keV)	PBFC							
	CuO_2_ wt%		CuO_4_ wt%		CuO_6_ wt%		CuO_8_ wt%	
	HVL	MFP	HVL	MFP	HVL	MFP	HVL	MFP
55	0.195	0.281	0.189	0.273	0.196	0.283	0.169	0.244
66	0.287	0.415	0.234	0.338	0.276	0.398	0.227	0.328
77	0.270	0.390	0.246	0.354	0.257	0.370	0.260	0.375

The HVL for photon energy 77 keV observed is in the range from 0.246 cm up to 0.270 cm, for comparison with HVL from previous reported references, can be seen in Table 3. The Pb/cement for photon energy 88 keV shows the lowest HVL of 0.004 cm^24^. Followed by Glass/cement/fly ash/sand for photon energy 140 keV with HVL 0.11 cm^25^. For energy around 55 keV, the HVL is 0.169 cm which lower than that of Bi_2_O_3_/natural rubber of 0.2 cm and Polysiloxane rubber filled/acetates/ carboxylates/BiF_3_/ ThF_4_/metal/fluorides about 0.21 cm^31,32^. This proves that the composite PBFC in this study shows high potentials for shielding application.

**Table 3:** Half value layer (HVL) in this study and the previous published references for comparison.

Materials	Energy (keV)	HVL (cm)	References
Pb/cement	80	0.004	^24^
Glass/cement/fly ash/sand	140	0.11	^25^
Bi_2_O_3_/natural rubber	50	0.2	^31^
Polysiloxane rubber filled/acetates/carboxylates/BiF_3_/ThF_4_/metal/fluorides	45	0.21	^32^
Portland cement/BaSO_4_/Fe_3_O_4_/CuO	55	0.169	Present study
	66	0.227	
	77	0.260	

XCOM is database by the theoretical calculation for the physical phenomena in the form of linear and mass attenuation coefficient, and cross section of photoelectric scattering, coherent and incoherent scattering, and pair production for shielding materials. We extend the photon energies for theoretical of composite in this study up to 10^8^ keV as can be seen in Fig. 3a. The physical phenomena show the cross section for energy 100 keV is similar and some of these phenomena only occurred at high energy ex. pair production^61–63^. For the photoelectric absorption, the incident photon is fully absorbed consequently photon energy use for kicking out of some electrons in the K-shell^24^.

## Conclusion

The structural properties of PBFC composites for X-ray radiation shielding applications were determined based on XRD and FTIR spectra. FTIR spectra shows several bonds are formed in the sample, namely: Fe–O, Ba–Fe–O, Ba–Fe–O, C–O–C, O–H. Values of the linear and mass attenuation coefficient, atomic and electronic cross section shows best agreement with theoretical calculation (XCOM) database for the photon energy < 77 keV. The effectiveness of radiation shield indicated by the lowest HVL and MFP indicated that composite with 8 wt% CuO. Composite in this study shows better values compared with the previous reported using composite rubber-based, indicated high potentials composite in this study for design new and efficient radiology room.

## Data Availability

The datasets used and/or analyzed during the current study are available from the corresponding author upon reasonable request.
